# EGFRvIII/integrin β3 interaction in hypoxic and vitronectinenriching microenvironment promote GBM progression and metastasis

**DOI:** 10.18632/oncotarget.6730

**Published:** 2015-12-22

**Authors:** Zhaoyu Liu, Lei Han, Yucui Dong, Yanli Tan, Yongsheng Li, Manli Zhao, Hui Xie, Huanyu Ju, He Wang, Yu Zhao, Qifan Zheng, Qixue Wang, Jun Su, Chuan Fang, Songbin Fu, Tao Jiang, Jiaren Liu, Xia Li, Chunsheng Kang, Huan Ren

**Affiliations:** ^1^ Department of Immunology, Harbin Medical University, Harbin 150081, China; ^2^ Department of Neurosurgery, Tianjin Medical University General Hospital, Tianjin 300052, China; ^3^ College of Fundamental Medicine, Hebei University, Baoding 071000, China; ^4^ College of Bioinformatics, Harbin Medical University, Harbin 150081, China; ^5^ Laboratory of Neuro-Oncology, Tianjin Neurological Institute, Tianjin 300052, China; ^6^ Department of Neurosurgery, The Oncology Hospital affiliated to Harbin Medical University, Harbin 150081, China; ^7^ Department of Neurosurgery, The Hospital affiliated to Hebei University, 071000 Baoding, China; ^8^ Laboratory of Medical Genetics, Department of Biology, Harbin Medical University, 150081 Harbin, China; ^9^ Department of Neurosurgery, Beijing Tiantan Hospital, Capital Medical University, Beijing, 100050, China; ^10^ Department of Anesthesia, Boston Children's Hospital and Harvard Medical School, Boston, MA, 02115, USA

**Keywords:** tumor microenvironment, EGFRvIII, integrin β3, glioblastoma, cilengitide^®^

## Abstract

Glioblastoma (GBM) is one of the most lethal brain tumors with a short survival time. EGFR amplification and mutation is the most significant genetic signature in GBM. About half of the GBMs with EGFR amplification express a constitutively autophosphorylated variant of EGFR, known as EGFRvIII. Our *in vitro* data demonstrated further enhanced EGFRvIII activity and tumor cell invasion in the tumor microenvironment of hypoxia plus extracellular matrix (ECM) vitronectin, in which EGFRvIII and integrin β3 tended to form complexes. The treatment with ITGB3 siRNA or the integrin antagonist cilengetide preferentially interrupted the EGFRvIII/integrin β3 complex, effectively reduced tumor cell invasion and activation of downstream signaling effectors. Cilengitide is recently failed in Phase III CENTRIC trial in unselected patients with GBM. However, we found that cilengitide demonstrated efficacious tumor regression via inhibition of tumor growth and angiogenesis in EGFRvIII orthotopic xenografts. Bioinformatics analysis emphasized key roles of integrin β3, hypoxia and vitronectin and their strong correlations with EGFRvIII expression in malignant glioma patient samples *in vivo*. In conclusion, we demonstrate that EGFRvIII/integrin β3 complexes promote GBM progression and metastasis in the environment of hypoxia and vitronectin-enrichment, and cilengitide may serve as a promising therapeutics for EGFRvIII-positive GBMs.

## INTRODUCTION

Glioblastoma (GBM, grade IV astrocytoma) is the most malignant brain tumor of adults. Epidermal growth factor receptor (EGFR) is amplified in approximately 50% of GBMs. Half of the gene amplification is associated with a type III variant of EGFR mutation that lacks exons 2–7 (EGFRvIII), leading to constitutively active signaling pathways that correlate with worse prognosis [1]. EGFRvIII-mediated signaling contributes to proliferative advantages, inhibits apoptosis and favors tumor cell invasion. Therefore, it becomes an important therapeutic target for kinase inhibitors, immunotoxins, and peptide vaccines, including Gefitinib, Erlotinib and Cetuximab [2, 3]. However, present applications of EGFR inhibitors fail to realize therapeutic expectations because nearly all patients become resistant to further treatment. Moreover, how EGFRvIII signaling promotes the interaction between tumor cells and their microenvironment to advance rapid progression is largely unknown [4, 5].

The complexity of tumor environment poses a great challenge to the development of therapy for GBM. Besides stromal cells, hypoxia and ECM are predominant components in GBM microenvironment, play eseential roles in glioma invasion and angiogenesis. It is reported that hypoxia contributes to migration and invasion of cancer cells by activating its master regulator, hypoxia inducible factor 1 (HIF-1) [6]; tumor cells adhere to ECM via integrin receptors and activate focal adhesion signaling, before locally degrade the ECM, creating a pathway to invade into adjacent tissues [7]. Among these ECM, fibronectin (FN) and vitronectin (VTN) were identified within primary brain tumors and upregulated within both the tumor-stroma and at advancing edge within brain parenchyma [8]. Several studies found both hypoxia and vitronectin may induce the recruitment of integrin αvβ3 to cell membrane of GBM cells, thereby activating the focal adhesion kinase (FAK) to promote tumor invasion [9, 10]. Recent evidence suggests that integrins are coupled to growth factor receptors in the regulation of multiple biological functions. Integrin β3 interacts with VEGFR2 in a phosphorylation dependent manner, which underlies a number of more complex responses, including thrombosis and pathological angiogenesis [11]. Ehlers–Danlos syndrome (EDS) cells via the integrin αvβ3–EGFR complexes, engage a paxillin-mediated pathway of cell survival [12]. As integrin αvβ3 is abundantly present on endothelial cells, research on this integrin receptor mainly focuses on its role in tumor angiogenesis and epithelial-to-mesenchymal transition. Recently, increased expression of integrin αvβ3 in GBM cells is reported as a poor prognostic factor [13].

Cilengitide is a novel integrin antagonist for the treatment of GBM and proved its effect in preclinical models at early clinical trials. However, recent data reveal that, the Phase III CENTRIC trial rules out any role for cilengitide in addition to standard treatment in GBM [14, 15]. Here we show in GBM cells, EGFRvIII was actively involved in a focal adhesion complex, which represents core components of integrin pathway and tumor cell invasion [16]. By construction of relevant EGFRvIII-expressing GBM cells, we show that, in the environment of hypoxia and vitronectin-enrichment *in vitro*, integrin β3 interacts directly with EGFRVIII and activates a SRC/FAK/EGFRvIII signaling axis to promote GBM cell invasion. More importantly, we demonstrate that targeted therapy with integrin αvβ3 antagonist cilengitide confirms better inhibition efforts in EGFRvIII-tumors. To our best knowledge, this is the first report to demonstrate that tumor environment contributes to induction of EGFRvIII signaling via integrin β3. Thus, we suggest further clinical testing with cilengitide in fractionated tumors of EGFRvIII-positive.

## RESULTS

### EGFRvIII-expressing GBM cells respond to the microenvironment of hypoxia and vitronectin by enhanced cell invasion

Bioinformatics analysis of the gene expression profiling of U87MG GBM cells that express EGFRvIII and vector control [16] suggested highly responsiveness of the mutant receptor to the environment. For example, *response to oxygen level* was the sixth significant GO (gene ontology) of the biological processes (*p = 3.30E*–*06*); *extracellular structure organization* the second GO (*p = 8.50E*–*07*), whereas *ECM-receptor interaction* and *focal adhesion* conferred to the second (*p = 1.51E*–*03*) and third (*p = 3.08E-03*) significant KEGG pathway enrichment, respectively. Other significant GO and pathways showed that the mutant receptor was highly relevant to cell motion, migration, inflammatory response and angiogenesis (Supplementary Figure S1A and 1B) [16]. Consistently, the gene expression data (Supplementary Figure S1C) and real-time RT-PCR verification (Supplementary Figure S1D) showed that, the expression levels of quite a few ECM components and integrins were significantly changed [16].

**Figure 1:**
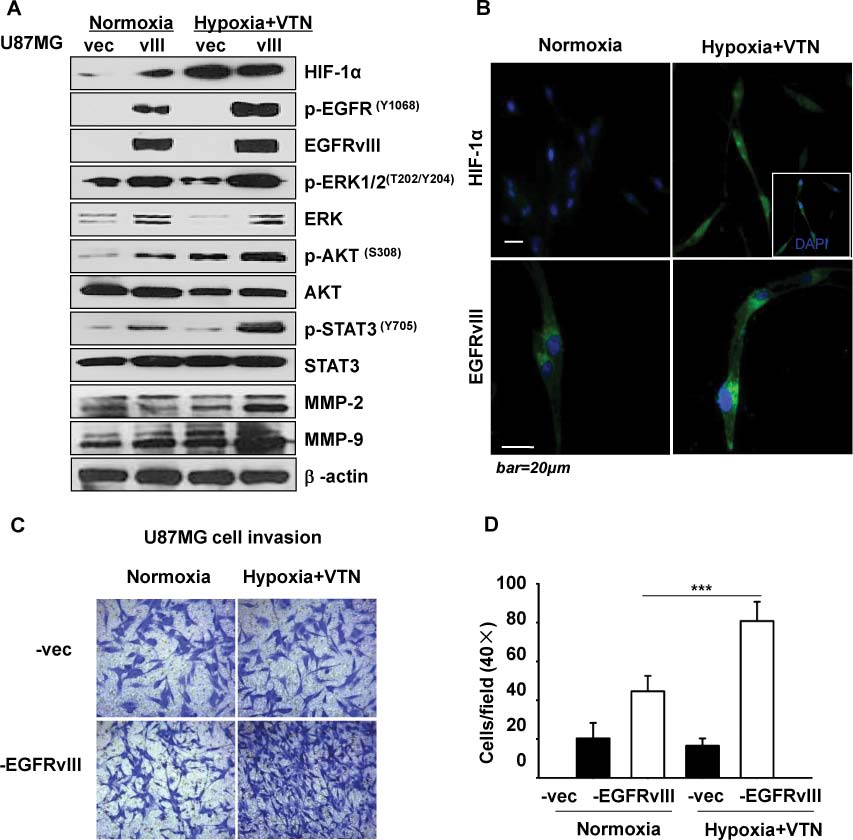
Effect of hypoxia and vitronectin microenvironment on U87MG-EGFRvIII cells (**A**) The specific microenvironment promoted significant activation of EGFRvIII and downstream signaling in U87MG-EGFRvIII but not vector cells. (**B**) Immunofluorescence showed greatly enhanced p-EGFRvIII expression in response to a hypoxia & vitronectin (VTN) cell cultural conditions in U87MG-EGFRvIII cells. (**C**) U87MG-EGFRvIII cells had significantly more enhanced invasion in response to the microenvironment than vector cells by Transwell invasion assays. (**D**) Quantification of the cell invasion rates in (C). Values are the mean and SEM of 5 independent determinations; data are representative of at least 3 independent experiments. ***p < 0.001.

To find out which ECM may be highly relevant to further EGFRvIII activation, we applied respective four different ECM components, i.e. collagen I or IV, laminin, fibronectin or vitronectin, in addition to a hypoxic condition, to the cell culture of U87MG clones, setting normoxia & non-ECM microenvironment as the control (data not shown). The results showed that, the microenvironment of hypoxia and vitronectin, but not other ECM, the most significantly enhanced activation of EGFRvIII and its downstream signaling effectors, i.e. ERK1/2 (MAPK), AKT and STAT3, and the expression level of the tumor cell invasion markers, i.e. matrix metalloproteinase-2 (MMP2) and MMP9 [16]. In contrast, the same conditional culture less activated these molecules in vector cells (Figure 1A). The data were confirmed in LN229 clones (Supplementary Figure S2A). Furthermore, immunofluorescence showed that the microenvironment greatly increased the expression of EGFRvIII, especially in trailing edge and protrusion of the filopodia in U87MG-EGFRvIII cells (Figure 1B). In Transwell assays, U87MG-EGFRvIII cells showed significantly higher invasion rates than vector cells under normal culture conditions. In comparison, U87MG-EGFRvIII cells in the environment of hypoxia and vitronectin-enrichment showed an 82% increment of cell invasion (Figure 1C and 1D). Besides, with or without hypoxic & vitronectin, we find little differences on long-term proliferation, for which U87MG-EGFRvIII cells were plated at a low density and counted daily until day 14 (data not shown). These data showed that the GBM cells under the microenvironment showed more active EGFRvIII signaling and invasive behaviors.

### Integrin β3 activates and interacts with EGFRvIII under the microenvironment

**Figure 2:**
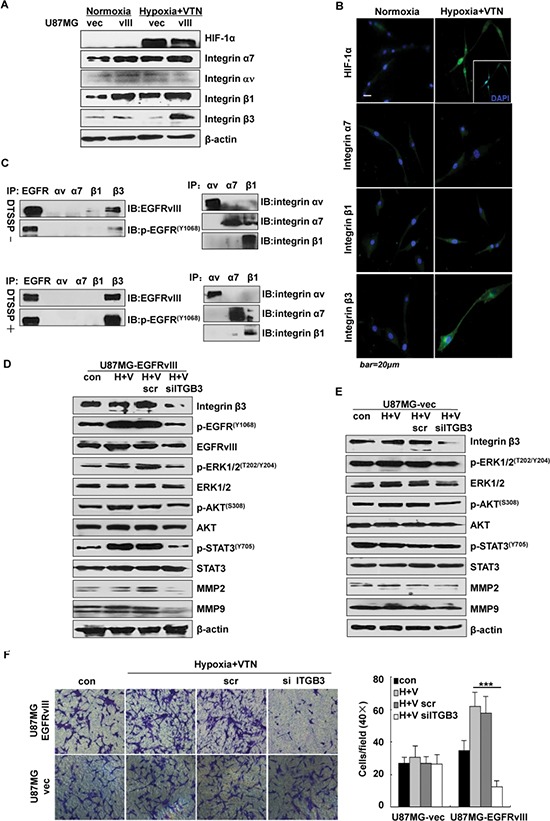
Integrin β3 regulated EGFRvIII activation under the microenvironment Greatly increased expression of integrin β3 in U87MG-EGFRvIII cells only in the environment of hypoxia and vitronectin-enrichment by western blotting assays (**A**) and in fluorescent microscopy (**B**). Note HIF-1α, integrin α7, integrin β1, integrin β3 (green), nuclear DNA (blue).(**C**) Cross-linking of EGFRvIII and integrin β3 with or without the membrane-impermeable cross-linker DTSSP in U87MG-EGFRvIII cells, the cross-linkage was not seen with EGFRvIII and other integrins. (**D**) *ITGB3* siRNA treatment in U87MG-EGFRvIII (D) but not vector cells (**E**) significantly reduced activation of EGFRvIII and downstream signaling effectors including the expression levels of MMP2 and MMP9 by western blotting assays. (**F**) *ITGB3* siRNA treatment significantly reduced cell invasion of U87MG-EGFRvIII but not vector cells by a 24 h Transwell invasion assay. Values are the mean ± SEM of 3 independent determinations; data are representative of 3 independent experiments. ***p < 0.001.

The receptors for vitronectin included the integrin αvβ1, αvβ3 and αvβ5 [17], among which, *ITGA3*, *ITGA7* and *ITGB3* were significantly up-regulated upon EGFRvIII expression in U87MG cells (Supplementary Figure S1C and 1D). Based on these data, we measured the expression of integrins αv, α7, β1 and β3 in U87MG clones with or without hypoxic & vitronectin. Western blotting data showed that, only the expression of integrin β3 was markedly increased in U87MG-EGFRvIII cells under the conditions (Figure 2A). Consistently, the immunofluorescent data showed more integrin β3 at the cell membrane under the microenvironment (Figure 2B).

To test if there were physical interactions between these integrins and EGFRvIII under the microenvironment, an assay with DTSSP, a chemical cross-linker, was performed in U87MG-EGFRvIII cells. Then the cell lysate was precipitated with anti-integrins αv, α7, β1 or β3 mAb, followed by immunoblotting with the anti-EGFRvIII total and Tyr1068 mAb. The results showed that, whereas positive co-precipitation of EGFRvIII or p-EGFRvIII with integrin β3, but not with other integrins, was observed without DTSSP, such co-precipitations were more significant in the cells treated with DTSSP (Figure 2C). Thus, we considered that EGFRvIII may be physically associated with integrin β3 in U87MG cells under the microenvironment. As the cross-linker DTSSP does not penetrate through the cell membrane [18] and only links with the cell surface proteins, these data also indicated that EGFRvIII and integrin β3 may form complexes associated with the cell membrane.

To determine whether integrin β3 increase may influence EGFRvIII signaling and cell invasion, U87MG cells were transfected with a small interfering RNA (siRNA) on *ITGB3* under the microenvironment. After 24 h, silencing of *ITGB3* resulted in decreased p-EGFRvIII at Tyr1068, but unchanged total EGFRvIII; in addition to greatly reduced activation of ERK1/2 (MAPK), AKT and STAT3, and expression of MMP2 and MMP9 (Figure 2D). By contrast, these molecules were less inhibited in vector cells under the treatment (Figure 2E). Moreover, Transwell assays showed that the siRNA treatment in U87MG-EGFRvIII, but not vector cells, greatly reduced cell invasion by 82% (Figure 2F).

### Integrin β3 induces stable activation of EGFRvIII via forming complexes with EGFRvIII and preventing its down-regulation

**Figure 3:**
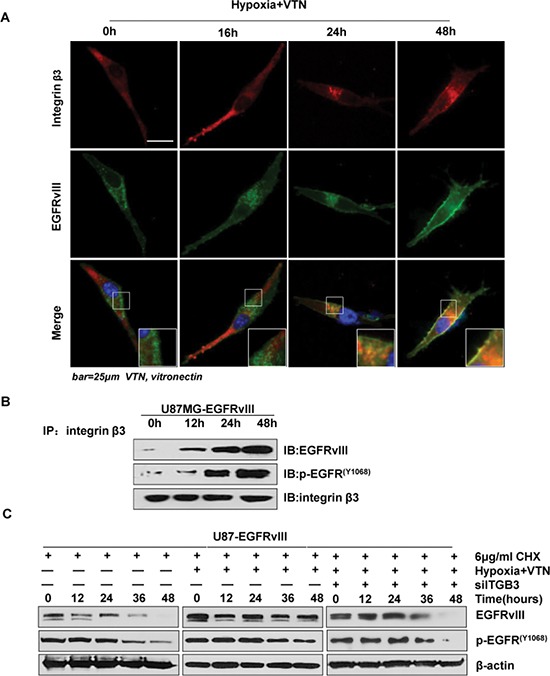
Integrin β3 associated with EGFRvIII and prevented its downregulation (**A**) Immunofluorescence indicated a time dependent association of integrin β3 and EGFRvIII from 24 h in U87MG-EGFRvIII cells in the environment of hypoxia and vitronectin-enrichment. (**B**) A time dependent association of integrin β3 and EGFRvIII by co-immunoprecipitation assays in U87MG-EGFRvIII cells under the microenvironment. (**C**) Sustained protein degradation rates of EGFRvIII and p-EGFRvIII in U87MG-EGFRvIII cells under the microenvironment, in comparison to relatively reduced rates under *ITGB3* siRNA treatment. Note, 6 ug/ml CHX was used.

To find out mechanisms of integrin-β3-mediated EGFRvIII activation under the conditions in GBM cells, we first tested the intracellular co-localization of integrin β3 and EGFRvIII in U87MG cells by confocal microscopy. The extended incubation under the microenvironment resulted in stronger co-localization of integrin β3 and EGFRvIII at the cytoplasm at 24 h and the spreading into the leading edges of cells at 48 h, and in parallel, a time-dependent activation of both molecules (Figure 3A). Double staining with both antibodies showed that integrin β3 did not co-localize with EGFRvIII under normal conditions or until 16 h of incubation under the conditions. At 48 h, the co-localization pattern was more obvious at the filopodia and lamellipodia of the cells. Overall, these showed a time-dependent increase of the integrin β3 and EGFRvIII complexes under the conditions. Co-immunoprecipitation assays confirmed an increased binding of EGFRvIII, especially in its activated form, with integrin β3 over time (Figure 3B).

We next examined if such a microenvironment triggered accumulation of integrin β3 may inhibit EGFRvIII degradation. To verify this, we evaluated the levels of EGFRvIII and p-EGFRvIII at Tyr 1068 by western blotting in the presence of the protein synthesis inhibitor CHX for up to 48 h. The results showed that, in the presence of CHX, whereas EGFRvIII and p-EGFRvIII at Tyr 1068 were gradually degraded without the microenvironment, the expression levels of both kept constitutively high throughout the time under the conditions. Importantly, both levels decreased rapidly after *ITGB3* was silenced by siRNA treatment (Figure 3C). Collectively, these data showed that, an increase of integrin β3 triggered by hypoxic & vitronectin formed complexes with EGFRvIII and prevented degradation of the mutant receptor, leading to persistent activation of EGFRvIII and downstream signaling, and greatly increased cell invasion.

### Integrin β3 is a key target of the integrin β3/FAK/SRC/EGFRvIII signaling axis on tumor cell invasion

**Figure 4:**
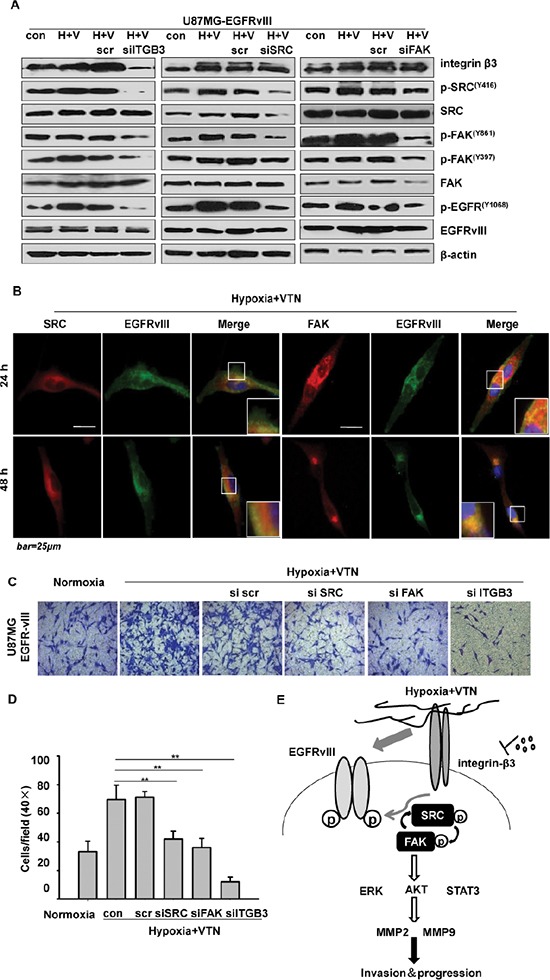
The integrin β3/FAK/SRC/EGFRvIII signaling axis (**A**) *ITGB3* siRNA treatment (left) the most significantly reduced the activation of p-EGFRvIII in comparison to the treatment with *SRC* siRNA (middle), or *FAK* siRNA (left) in U87MG-EGFRvIII cells under the microenvironment by western blotting assays. (**B**) Immunofluorescence indicated the association of EGFRvIII and FAK, or SRC, in U87MG-EGFRvIII cells under the microenvironment. (**C**) *ITGB3* siRNA treatment the most significantly reduced the cell invasion in U87MG-EGFRvIII cells by Transwell invasion assays, as compared with *SRC*, or *FAK* siRNA treatment. (**D**) The quantitative analysis of invasive U87MG-EGFRvIII cells treated with *FAK*, *SRC*, or *ITGB3* siRNA. The data are shown as the mean ± SEM of at least 3 independent experiments. ***p < 0.001. (**E**) Diagram: Crosstalk of integrin β3 with EGFRvIII and an integrated integrin β3/FAK/SRC/EGFRvIII signaling axis in promoting tumor cell invasion and progression in EGFRvIII-expressing GBM cells in the environment of hypoxia and vitronectin-enrichment.

We previously showed that, EGFRvIII was actively engaged in focal adhesion complexes and associated signaling to promote GBM cell invasion [16]. Formation of FAK-SRC complexes was a focal point for focal adhesion signaling and cell migration [19, 20]. We then tested if activity of FAK and/or SRC was reduced in U87MG-EGFRvIII cells by *ITGB3* siRNA treatment under the microenvironment. The siRNA treatment greatly reduced not only p-EGFRvIII at Tyr1068, but also p-FAK at Tyr397, which is the site of FAK autophosphorylation upon engagement of integrins and the ECM ligands; and at Tyr861, which is the active site for the following SRC phosphorylation [21], as well as p-SRC (Figure 4A left). To compare the effect among targeted therapies at each of these focal adhesion-associated molecules on EGFRvIII activity, and active interactions among them, further siRNA assays were used. The results showed that, siRNA treatment on *SRC*, or *FAK*, in the GBM cells reduced the level of p-EGFRvIII (Figure 4A middle and right), yet to a less extent than that of *ITGB3* siRNA (Figure 4A). Moreover, whereas siRNA treatment on *SRC* or *FAK* did not reduce the expression of integrin β3, the treatment with *SRC* siRNA reduced the level of p-FAK at Tyr861, yet to a less extent than that by *ITGB3* siRNA (Figure 4A middle and left); in comparison, the *FAK* siRNA only slightly reduced p-SRC level (Figure 4A right).

We further verified that, EGFRvIII directly associated with SRC and/or FAK in the GBM cells, and thus, located within the focal adhesion complexes in the environment of hypoxia and vitronectin-enrichment by immunofluorescence. There existed stronger co-localization of EGFRvIII with FAK than with SRC during the time in U87MG cells (Figure 4B). Transwell assays were used to assess relevant functions of integrin β3, SRC or FAK in U87MG-EGFRvIII cells. Whereas transfection of the GBM cells with *SRC* or *FAK* siRNA led to respective 40% and 49% inhibition as compared to non-silenced cells under the microenvironment, targeted inhibition on *ITGB3* achieved the most effective inhibition on cell invasion by 83% (Figures 2F, 4C and 4D). Taken together, we showed that, the tumor microenvironment activated an integrin β3/FAK/SRC/EGFRvIII signaling axis, where integrin β3 was the most important regulator on modulating EGFRvIII activity and GBM cell invasion *in vitro* (Figure 4E).

### Functional interaction of integrin β3 and EGFRvIII is significant in EGFRvIII-expressing GBM *in vivo*

**Figure 5:**
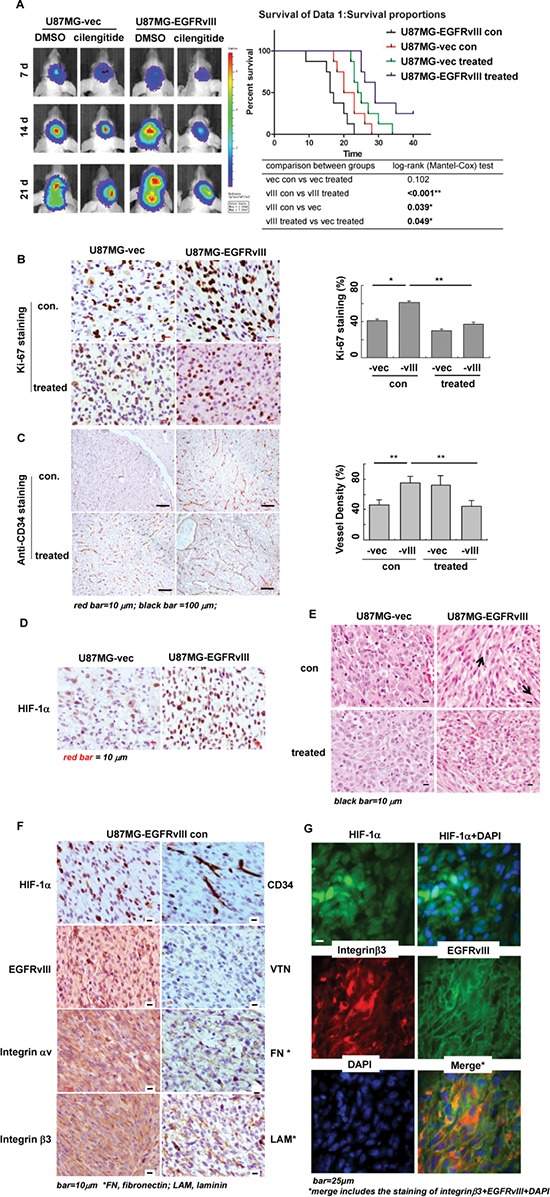
Tumor microenvironment induced by EGFRvIII in GBM xenografts *in vivo* (**A**) The tumor burdens at different time points *in vivo* by bioluminescent imaging; and analysis on the survival rates in U87MG-vector or -EGFRvIII-bearing animals with/out the treatment with cilengitide^®^. Tumor cell proliferation with Ki-67 (**B**), tumor angiogenesis and microvascular density (MVD) with CD34 (**C**), and areas of hypoxia with HIF-1α (**D**) staining by immunohistochemistry (IHC) in U87MG-vector and -EGFRvIII xneografts with/out treatment. (**E**) The bundle-like cell groups in non-treated EGFRvIII-tumors by Hematoxylin-eosin (HE) staining, in comparison to the tumor in other three groups. The arrows in non-treated EGFRvIII-tumor group indicated directions of the bundle-like cell groups. (**F**) High expression levels of HIF-1α, CD34, EGFRvIII, integrins αv and β3, and the ECM components, vitronectin (VTN), fibronectin (FN), laminin (LAM) by IHC in the bundle-like tumor cell groups, as shown in (E) in non-treated EGFRvIII-tumors. (**G**) The expression of HIF-1α (green), integrin β3 (red) and EGFRvIII (green) by immunofluorescenc in the bundle-like tumor groups in non-treated EGFRvIII-tumors. Tumor samples for IHC and HE staining were taken 15 d after tumor cell implantation. All experiments were performed independently at least two times. *p < 0.05; **p < 0.01.

To analyze a tumor microenvironment that induced by EGFRvIII in GBM *in vivo*, we used GBM orthotopic animal models that were implanted with U87MG cells [16]. The data showed that, as compared to vector xenografts, –EGFRvIII tumors exhibited a greatly shortened survival (Figure 5A), characteristics of a more advanced tumor grade, including a higher tumor proliferation rate as shown by Ki-67 staining (Figure 5B), intensive angiogenesis with a great number of necrotic foci and aggressive tumor invasion to adjacent tissues (Figure 5C, 5D and Supplementary Figure S2B). These data kept consistent with our bioinformatics analysis on differential expression profiles between U87MG-EGFRvIII and -vector cells *in vitro*, which highly suggested potentials of the mutant receptor to induce such a microenvironment (Supplementary Figure S1A and 1B) [16].

Furthermore, whereas the tumor morphological features and structures were distinct between EGFRvIII-expressing GBM and vector controls, crisscrossed bundle-like tumor cell groups were commonly observed in EGFRvIII-tumors (Figure 5E), assuming each functioning as an invading block. These cell blocks were not only intensively stained with the marker for hypoxia, HIF-1α, but also contained a number of budding and extended vessels (Figure 5F, Supplementary Figure S2B, anti CD34 staining). Moreover, expression levels of EGFRvIII, integrin αv, β3, and ECM components, i.e. vitronectin, fibronectin and laminin, were much rich in these blocks (Figure 5F). Moreover, we showed a strong co-lolization of EGFRvIII and integrin β3 by immunofluorecent microscopy in these bundle-like tumor cells *in vivo* (Figure 5G).

**Figure 6:**
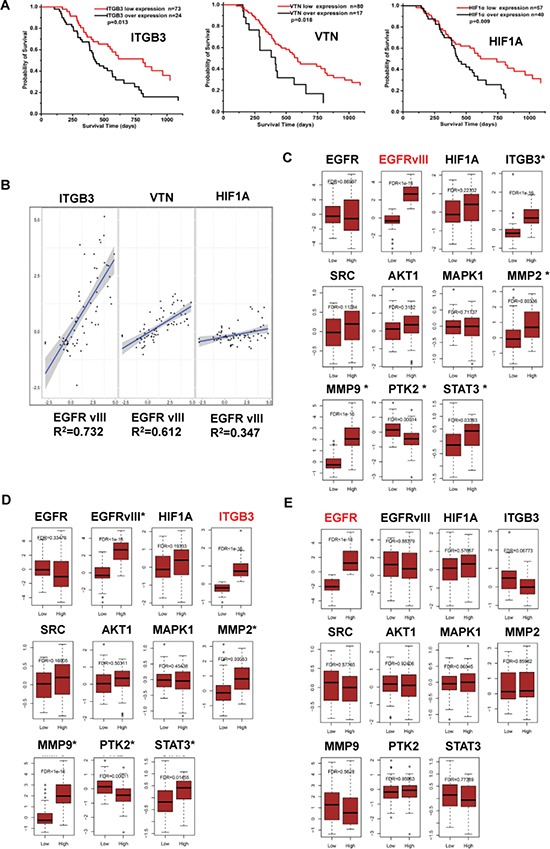
Significance of EGFRvIII, integrin β3, HIF-1α, VTN, and their relations in malignant glioma samples (**A**) Kaplan-Meier analyses [38] on the correlations between the relative expression of *ITGB3*, *HIF-1α*, or *VTN* (vitronectin), and the overall survival time of 97 patients with malignant glioma at the mRNA level. (**B**) Pearson correlation on the transcriptional level of *ITGB3*, *HIF-1α*, or *VTN*, and *EGFRvIII* in these tumor samples was analyzed by R software. Significance of *EGFRvIII*, *ITGB3* and *EGFR* in these tumor samples were analyzed by bioinformatics methods as indicated in Materials and Methods. In the gene sets regulated by *EGFRvIII* (labeled in red) (**C**), or *ITGB3* (**D**), or *EGFR* (**E**); effector expressions of the integrin β3/EGFRvIII-associated signaling axis in Figure 4E were shown; the effectors that had a significant change in gene expression were labeled with *.

Bioinformatics analysis was used to verify significance of these factors in GBM biology on cDNA array data of tumor biopsies from 97 patients, including 33 Grade III glioma and 64 GBM [22]. Kaplan-Meier analysis showed that, respectively higher expressions of *ITGB3*, *VTN* (vitronectin) or *HIF1A* was significantly correlated with shortened survival rates in these patients (Figure 6A, *p* < 0.05). The level of EGFRvIII expression was greatly correlated to that of *ITGB3*, *VTN* or *HIF1A*, respectively (Figure 6B). Further comparisons on *EGFRvIII* and *ITGB3* regulated gene sets by respective GO and KEGG pathway enrichment analysis showed that, whereas these two gene expression mutually interacted, a few other genes that were involved in focal adhesion and invasion stood out in both sets, including *PTK2* (FAK), *STAT3*, *MMP2*, *MMP9* etc.(Figure 6C and 6D). Consistently, “focal adhesion” and “ECM-receptor interaction” showed in the list of significant pathways that were regulated by *EGFRvIII* or *ITGB3* (Table 1). In contrast, the wild type receptor *EGFR* did not attain such effects (Figure 6E and Table 1). Collectively, these data elucidated functional interactions between integrin β3 and EGFRvIII in a real tumor microenvironment, and EGFRvIII-driven properties in patients with malignant glioma.

**Table 1:** The KEGG pathways that significantly enriched by genes regulated by *EGFRvIII, ITGB3* or *EGFR*

EGFRvIII	*p* value	ITGB3	*p* value	EGFR	*p* value
neuroactive ligand receptor interaction	< *.001*	valine leucine and isoleucine degradation	< *0.01*	TGF beta signaling pathway	< *.001*
propanoate metabolism	< *.001*	terpenoid backbone biosynthesis	< *.001*	hedgehog signaling pathway	< *.001*
valine leucine and isoleucine degradation	< *.001*	cytokine cytokine receptor interaction	< *.001*	basal cell carcinoma	< *.001*
ECM receptor interaction*	< *.001*	propanoate metabolism	*.001*	dorso ventral axis formation	< *.001*
butanoate metabolism	*.001*	neuroactive ligand receptor interaction	*.001*	glycine serine and threonine metabolism	*.001*
terpenoid backbone biosynthesis	*.001*	beta alanine metabolism	*.001*	tryptophan metabolism	*.002*
oocyte meiosis	*.001*	butanoate metabolism	*.002*	pyruvate metabolism	*.002*
beta alanine metabolism	*.002*	cell adhesion molecules	*.003*	valine leucine and isoleucine biosynthesis	*.002*
cysteine and methionine metabolism	*.002*	cysteine and methionine metabolism	*.007*	lysine degradation	*.003*
cytokine cytokine receptor interaction	*.002*	ECM receptor interaction	*.007*	valine leucine and isoleucine degradation	*.003*
nitrogen metabolism	*.004*	limonene and pinene degradation	*.008*	terpenoid backbone biosynthesis	*.004*
limonene and pinene degradation	*.004*	oocyte meiosis	*.008*	glyoxylate and dicarboxylate metabolism	*.004*
focal adhesion	*.004*	leukocyte transendothelial migration	*.009*	mismatch repair	*.009*
pyruvate metabolism	*.006*	tryptophan metabolism	*.010*	protein export	*.010*
cell adhesion molecules	*.007*	pyruvate metabolism	*.010*	selenoamino acid metabolism	*.012*
maturity onset diabetes of the young	*.014*	nitrogen metabolism	*.011*	citrate cycle tca cycle	*.017*
tryptophan metabolism	*.015*	primary immunodeficiency	*.011*	ERBB signaling pathway	*.017*
lysine degradation	*.017*	renin angiotensin system	*.017*	propanoate metabolism	*.018*
leukocyte transendothelial migration	*.029*	focal adhesion	*.017*	GAP junction	*.019*
vibrio cholerae infection	*.036*	oxidative phosphorylation	*.022*	butanoate metabolism	*.019*

* The grey highlighted pathways are greatly relevant to focal adhesion complex-associated signaling.

### Treatment with cilengitide attained high efficacy in EGFRvIII-expressing GBM via breaking down integrin β3/EGFRvIII signaling axis

**Figure 7:**
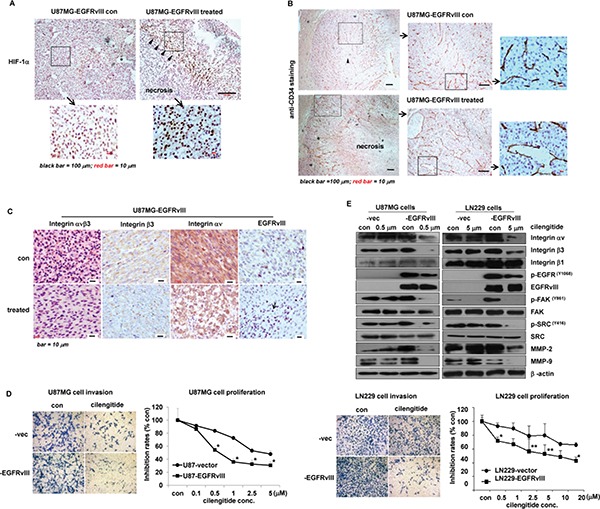
Cilengitide achieved high efficacy in treating EGFRvIII-tumors (**A**) Focal necrosis was commonly observed in non-treated EGFRvIII-tumors. *indicated the necrotic areas; the insert showed enlarged HIF-1α staining (the left panel). The treatment in EGFRvIII-tumors resulted in a huge necrotic area and a minor wave of tumor cell palisades between the necrotic area and normal brain tissues; Triangles indicated hypoxic tumor cells by strong positive HIF-1α staining (the right panel). (**B**) Comparison of tumor microvascular hyperplasia in EGFRvIII-xenografts with/out the treatment. From the left to right, the inserts pointed out details of the tumor vessels. The tumor vessels in non-treated tumors were complete and extending; the triangle indicated tumor necrosis in the center of the tumor burden (the upper panel); whereas that in the treated tumor showed acute broken-down of the vessel walls and vascular congestion and dilation. There was a huge tumor necrosis area as a result of the treatment (the lower panel). (**C**) The treatment significantly reduced the expression of integrin αvβ3, EGFRvIII, and cellular distribution of integrins αv and β3 by IHC. The arrow in the bottom right panel indicated the EGFRvIII-positive staining inflammatory cells in the treated tumor samples. (**D**) The treatment more significantly inhibited cell invasion and proliferation in EGFRvIII-expressing GBM than vector cells in the environment of hypoxia and vitronectin-enrichment *in vitro*. (**E**) The treatment more efficiently inhibited the expression of the integrin αν, integrin β3, p-EGFRvIII^Y1068 (145kD)^, p-FAK^Y861^, p-SRC^Y416^, MMP2, MMP9 etc. in EGFRvIII-expressing GBM than vector control cells. According to different IC50 in (D), respective 0.5 or 10 μM of cilengitide were used on U87MG- or LN229 clones for 24 h. Tumor samples *in vivo* were taken 15 d after tumor cell implantation. All experiments were performed independently at least three times. *p < 0.05; **p < 0.01.

To investigate efficacy of a targeted therapy against integrin β3 *in vivo*, we treated EGFRvIII and vec xenografts with the integrin αvβ3 antagonist cilengitide. The results showed that, the treatment achieved a high efficacy with greatly reduced proliferation rates of the tumor cells (Figure 5B), decreased density of the tumor vessels (Figure 5C and Supplementary Figure S2B), and improved survival rates (Figure 5A) in EGFRvIII but not vec-xenografts. In addition, the therapy with cilengitide also markedly disturbed key features of EGFRvIII-expressing GBM *in vivo*, leading to huge necrosis blocks in the tumor burden (Figure 7A). Especially, those bundle-like cell groups of the untreated tumor shrank into a minor wave of cell palisades along border of the huge necrosis tumor area, next to the normal brain tissues (Figure 7A). Furthermore, the treatment significantly disrupted organization of the tumor microvascular structure, and resulted in acute break-down of the tumor vessels (Figures 5C and 7B), in addition to acute inflammation (EGFRvIII staining in the treated group of Figure 7C). Moreover, the treatment significantly reduced expression levels of EGFRvIII, integrin αvβ3, VTN, as well as the cellular distributions of integrin β3, αv in non-necrotic area of the tumor mass *in vivo* in EGFRvIII-xenografts (Figure 7C and Supplementary Figure S2C). In contrast, the same therapy was less effective in treating U87MG-vector xneografts (Figure 5A–5C). Noticeably, however, the treatment with cilengitide attained an irritation effect on the tumor vessels, leading to significantly increased vascular density in vector tumors (Figure 5C and Supplementary Figure S2B-S2E). Collectively, these data showed high efficacy with cilengitide therapy in EGFRvIII-expressing GBM via disruption of the tumor microenvironment, angiogenesis and associated EGFRvIII activity *in vivo*.

Further data with cilengitide on GBM cells with/out EGFRvIII expression under the microenvironment, including a primary GBM culture, confirmed the drug efficacy on cell invasion and proliferation esp. in EGFRvIII-expressing cells (Figure 7D and Supplementary Figure S3). More importantly, besides the drug inhibition on tumor cell functions, we observed more significant inhibition on expressions of the drug targets, and activity of associated signaling molecules in EGFRvIII-expressing but not vector GBM cells, i.e. integrin αν, β3, p-EGFRvIII, p-FAK, p-SRC, MMP2, MMP9, etc. (Figure 7E and Supplementary Figure S2F). These data kept close consistency with those *in vivo*, emphasizing significance of coherent interactions of integrin β3 and EGFRvIII within the tumor microenvironment (Figure 2F).

## DISCUSSION

EGFR is one of the most important biomarkers for cancer molecular classification, yet may be not an ideal therapeutic target in GBM [23]. Thus far, the targeted therapy against EGFR or EGFRvIII demonstrates only limited efficacy in GBM patients, partially due to the fact that, constitutive activation of the receptor well establishes its network to drive tumor progression, where the receptor itself is not the best target [16, 23]. Active interaction between the tumor and environment and the intracellular signaling within the tumor cells play an essential role in such a network [1]. Here, we showed significance of functional interactions between EGFRvIII and integrin β3 via receptor cross-linking and mutual stimulation in formation of an integrin β3/FAK/SRC/EGFRvIII signaling axis, and in rapid tumor progression. In this scenario, integrin β3 was the major hub for both the inside-out and outside-in signaling, and thus, an ideal target to disturb the network of EGFRvIII expressing GBM for malignant progression.

During development, cells create their intrinsic environment by manipulating ECM components to support the advance of all structures that comprise a functioning organism [24]. Likewise, within the same brain environment of the orthotopic models established by tomor cells injection, U87MG-EGFRvIII cells rapidly created a much different tumor environment from vector cells to support more advanced tumor growth and progression [25]. Although both types of GBM were rich in ECM protein and highly vascularized, our data elucidated a superb ability and mechanisms of EGFRvIII to initiate and maintain integrated functions linking tumor cells and their surrounding environment, via forming complexes with integrin β3. Angiogenesis and invasion are undoubtedly critical features in GBM development and progression. The environment factors, i.e. ECM components, integrins and conjugated focal adhesion pathways contribute greatly to these properties of GBM [8]. Our series reports showed that, as one of the key components of the focal adhesion complexes, EGFRvIII takes more advantages of this pathway in GBM [16]. Furthermore, besides in GBM cells and animal models, we showed, in human malignant glioma biopsies [22], strong interactions between EGFRvIII and integrin β3 via coherent focal adhesion pathways, however such an interaction was not found between EGFR and integrin β3 in the same tumor samples. Therefore, in our reports, we elucidate distinct properties of EGFRvIII, different from that of the wild type EGFR, in GBM biology [16].

Cilengitide is an antagonist of integrins. However, The Phase III CENTRIC trial excluded any role for cilengitide plus standard treatment in newly diagnosed GBM [14, 15]; consistently, we observed only modest effects with the treatment in U87MG-vector GBM *in vivo* and *in vitro*. We thus suggest further clinical testing with cilengitide in EGFRvIII-positive tumors. Our data emphasize that, conjugated function of tumor microenvironment, i.e. hypoxia, ECM, integrins and the mutant EGFR, makes an integrated unit for rapid tumor progression, where the intergin β3 comprises the best target, as the integrin is not only expressed in tumor cells, but also in activated endothelial cells during tumor angiogenesis [26]. However, how interaction between EGFRvIII and integrin β3 in creating a microenvironment of intensive angiogenesis *in vivo* warrant further investigations. Indeed, a better understanding of the tumor microenvironment, and reasonable real-time tests on tumor profiles for selection of suitable patients could lead to more optimized therapies and avoid from resistance [27].

In conclusion, our data provide important evidence that, within the tumor microenvironment, integrin β3/EGFRvIII crosstalk is a key element driving tumor growth and progression; cilengitide can be a promising therapeutics for EGFRvIII-positive GBM.

## MATERIALS AND METHODS

### Cell culture

The GBM cell lines U87MG-, LN229-vector, and -EGFRvIII, were established as previously described and maintained in our laboratory [16]. Briefly, full-length cDNAs of EGFRvIII was amplified with PSK plasmids (kind gifts from Dr. F. Furnari, Ludwig Institute, San Diego, CA, USA) into the expression vector pcDNA3.1 (−) (Invitrogen, Carlsbad, CA, USA), then transfected into the GBM cells with Lipofectamine 2000 (Invitrogen). Stable GBM cell clones were initially selected using 600–800 μg/mL of the antibiotic G418 (EMD Biosciences, Shanghai, China) for 2–3 weeks. The cells were routinely maintained in DMEM supplemented with 10% fetal bovine serum, 1% penicillin/streptomycin and 400 μg/ml G418 at 37°C in 5% CO_2_ humidified incubators. Hypoxic conditions (2% O_2_, 5% CO_2_, 93% N_2_) were obtained by incubating the GBM cells in an anaerobic work station (ProOx C21, BioSpherix, USA).

### Antibodies and reagents

All antibodies were obtained from Cell Signaling Technology (CST, Danvers, MA, USA) unless indicated. The antibodies for integrin αv, integrin α7, integrin β1, integrin β3, and EGFR (wild type, for immunohistochemistry) were obtained from Santa Cruz Biotechnology (Santa Cruz, CA, USA). For IHC staining only, anti-EGFRvIII specific antibody was from Biosynthesis Biotechnology Co. Ltd., Beijing, China; others from ZSGB-Bio Co. Ltd., Beijing, China. Fluorescently labeled secondary antibodies and phalloidin were from Invitrogen (Courtaboeuf, France). Plasma vitronectin were from Sigma-Aldrich (StLouis, MO, USA). The integrin αvβ3 and αvβ5 antagonist cilengitide (Selleck Chemicals Co.Ltd, Shanghai, China) was dissolved in dimethyl sulfoxide (DMSO), aliquoted and stored at −20°C. Cell culture medium was used to serially dilute stock solutions to the required concentrations.

### Cell proliferation inhibition assays

Cell growth inhibition was performed using 48 h microplate MTT assays after the tumor cells experienced with a microenvironment of hypoxia and vitronectin, in which synchronized tumor cells were incubated in 0.1 μg/cm^2^ vitronectin-coated tissue culture plates for 6 h before subjected to hypoxia conditions for 24 h. The tumor cells were harvested and plated at a density of 2–3 × 10^3^ cells/well in microplates, treated with varying concentrations of cilengitide (0.1–5 μM on U87MG and 1–20 μM on LN229 clones). After 48 h incubation, cell medium were replaced by 0.5 mg/ml MTT (Sigma-Aldrich) and cells were incubated for further 4 h at 37°C. The intracellular formazan crystals were solubilized in 100 μl DMSO; Absorbance was measured at 570 and 630 nm on a microplate reader (SpectraMax M5, Molecular Devices, Sunnyvale, USA).

### Transwell invasion assays

Transwell chambers were used for cell invasion assays. Transwell filters (8 μm pore size, Corning, NY, USA) were pre-coated with Matrigel (BD Biosciences, San Jose, CA, USA) in a 24-well plate. A total of 2 × 10^4^ cells in 100 μL of serum-free medium were seeded on the upper side of the filters. The lower chamber was filled with 600 μL of medium containing 5% FBS. For hypoxia and vitronectin conditions, the upper chambers were pre-coated with 0.1 ug/cm^2^ of vitronectin on the Matrigel and incubated in anaerobic work station. After 24 or 48 h, cells migrating to the lower side were fixed, stained with 5% crystal violet (Sigma) and counted in 6 random fields under the light microscope.

### Western blotting analysis

Whole-cell lysates were harvested using cell lysis buffer (CST) with PMSF (Sigma), pepstatin A (Sigma), and PhosSTOP (Roche). Protein concentrations were determined by BCA assay (Sigma). Equal amounts of total protein were separated on an 8–10% SDS-polyacrylamide gel and electrophoretically transferred onto a nitrocellulose membrane (Millipore, Bedford, MA, USA). The membranes were blocked with Tris-buffered saline Tween-20 (TBST) containing 5% (w/v) skim milk for 1 h before incubating overnight at 4°C with the primary antibody. After washing with TBST, the membranes were incubated with a horseradish peroxidase-conjugated secondary antibody for 1 h at room temperature. The signals were visualized using enhanced chemiluminescence (Roche) and exposed to X-ray film.

### Immunoprecipitation assay and chemical cross-linking

GBM cells were plated into 10-cm tissue cultural dishes to reach 70% confluence, treated with ice-cold RIPA buffer (Sigma), and disrupted by repeated pipetting. For immunoprecipitation assays, 1 μg of antibody was added to 1 mg of protein lysate. After a 4 h-incubation at 4°C, 20 μL of protein A-agarose (Santa Cruz, CA, USA) was added, and the samples were incubated overnight at 4°C. After washing with cold RIPA buffer, the immunoprecipitated proteins were eluted for western blotting as described above. For cross-linking assays, cells were incubated with 1 mM DTSSP (3, 3′ dithiobis-[sulfosuccinimidylpropionate], Thermo Scientific, USA) in PBS at 4°C for 30 mins, followed by washing three times with TBS before used for further studies.

### siRNA transfection

Cells were transfected with varied siRNA (Genepharma, Shanghai, China), including a random sequence, siScramble, sense:(5′-UUCUCCGAACGUGUCACGUTT-3′); a siRNA specific to human *ITGB3*, sense:(5′-CCUGCACCUUUAAGAAAGATT-3′); a siRNA targeted to human *FAK* messenger, sense:(5′-GUGGAGGACUCUACAGUAUTT-3′); a siRNA targeted to human c-*SRC* messenger, sense:(5′-CGCGCCUCAUUAAACCAAATT-3′). Exponentially growing U87MG clones were transfected with the *ITGB3*, *FAK*, or *SRC* siRNA and Lipofectamine 2000 in the conditions recommended by the manufacturer (Invitrogen). The concentration of siScramble is similar to that of specific siRNA. Cells were used after 48 h with western blotting and adhesion assays as described above.

### Immunofluorescent microscopy

Standard immunostaining was performed. In brief, cells were grown on glass cover slips in 12-well plates before fixed with 4% paraformaldehyde. The cover slips were incubated in blocking solution, followed by incubation with the primary antibody at a 1:100 dilution overnight at 4°C. FITC-or Alexa fluor-labeled anti-rabbit or anti-mouse antibody was added to the incubation. The nuclei were stained with DAPI (Sigma). The samples were observed under a fluorescence microscope (Nikon, ECLIPSE 80i), or a ZEISS LSM 510 META confocal microscope (Carl Zeiss, Jena, Germany). The images captured by confocal microscope were using a 40× objective (Plan-Apochromat 40×/1.40 DIC M27) and processed using LSM Image browser software.

### Nude mouse GBM intracranial models and treatment

To construct intracranial GBM models [16], U87MG-vector and -EGFRvIII cells were infected with a luciferase lentivirus (Genepharma, Shanghai, China). After a 4-day infection, 5 × 10^5^ cells were collected and injected into the intracranial striatum of 5-week-old female Nu/Nu-Nude mice with a stereotactic instrument using a previously described guide-screw system. The animals were randomly divided into 4 groups, each with 11 mice. Starting on day 4 after tumor cell implantation, the mice were intraperitoneally injected with 4 mg/kg cilengitide in DMSO or with an equal volume of DMSO alone every other day until ten doses were given. To acquire tumor growth status in live animals with/out treatment by bioluminescent imaging, the mice were anesthetized, intraperitoneally injected with 50 mg/mL of D-luciferin (Promega, Shanghai, China), and imaged with the IVIS Imaging System (Caliper Life Sciences) for 10–120 s. Fifteen days after tumor cell implantation, 3 animals from each group were sacrificed, and the tumor samples were taken for IHC and HE staining. The remaining 8 mice in each group were used for survival analysis.

### Immunohistochemistry and evaluations

Immunohistochemistry with xneograft brain tissues was applied in 5 μm paraffin sections by a two-step method [16]. The primary antibodies including anti-EGFR, -EGFRvIII, -integrin αv, -integrin β3, -integrin αvβ3, -integrin β5, and -HIF-1α, -Ki-67, -vitronectin, -fibronectin, -laminin and -CD34 antibodies (each diluted at 1:100) were used. Negative controls were applied identically but with omission of the primary antibody. The positive staining in whole view was measured (Leica DM-IRE2, Cambridge, UK). Images of 6 representative fields at 400× magnifications were captured by Leica QWin Plus v3 software. Identical settings were used for each image. Proliferation index (PI) by Ki-67 staining was calculated. MVD (microvascular density) on anti-CD34 antibody staining was assessed [28]. Briefly, CD34-positive sections were initially scanned at low power (100×) and the areas with the highest neovascularization stained by the anti-CD34 antibody were chosen as hot spots. Microvessel assessment was carried out in five fields of the hot spots at 400× magnification. The brown-stained endothelial cells or cell cluster that were clearly separated from adjacent microvessels, tumour cells, and other connective tissue were identified as a single vessel. All experiments were performed independently at least 3 times and by a pathologist.

### Significance of EGFR, EGFRvIII and ITGB3 in human malignant glioma samples

A retrospective series of 97 glioma patients from Beijing Tiantan Hospital was considered. All samples were histologically graded according to current WHO classification on tumours of the nervous systems, including 33 grade III patients and 64 GBM patients. Written informed consent was obtained from all donors. Genome-wide mRNA expression profiling was obtained from these samples by Agilent Whole Human Genome Array. The gene expression were background-subtracted, quantile-normalized and log2-transformed. Nonspecific probes were removed; the expression of the redundant probes was averaged if multiple probes corresponded to a single gene [22].

To identify genes that were respectively regulated by *EGFR*, *EGFRvIII* or *ITGB3* in glioma, the samples were sorted into two resulted groups based on the median expression level of *EGFR* (*EGFRvIII* or *ITGB3*). *T*-test was used to recognize differentially expressed genes between these two groups. Genes with P-adjusted less than 0.05 were regarded as those regualted by *EGFR* (*EGFRvIII* or *ITGB3*). KEGG Pathway Enrichment Analysis was further performed in respective gene sets regulated by *EGFR*, *EGFRvIII* or *ITGB3*. If the whole genome had a total of N genes, of which K were involved in the pathway under investigation, and the set of target genes for analysis had a total of M genes, of which x were involved in the same pathway, then the *P* value can be calculated to evaluate the enrichment significance for that pathway as follows:

$$p=1-F(x|N,K,M)=1-\sum_{t=0}^{x}\frac{\left(\begin{array}{c} K\\ t \end{array}\right)\left(\begin{array}{c} N-K\\ M-t \end{array}\right)}{\left(\begin{array}{c} N\\ M \end{array}\right)}$$

Pathways with *P* values < 0.01 were regarded as significant pathways, which were sorted by the number of genes annotated in each pathway. All data were deposited in Chinese Glioma Genome Atlas (CGGA), a database on human glioma.

### Statistical analysis

Survival was analyzed by a log-rank test based on the Kaplan–Meier test using Origin 8 software (originpro.software.informer.com) in xenografts and X-tile in human malignant glioma samples [29]. R Software (r-project.org) was used for Pearson correlation analysis. Student's *t*-test was performed to compare the means of values from different experiments. Differences were considered statistically significant at *p* < 0.05*, < 0.01**, and < 0.001***.

## SUPPLEMENTARY MATERIAL AND METHODS FIGURES


