# Supplementary material for: EGFRvIII/integrin β3 interaction in hypoxic and vitronectinenriching microenvironment promote GBM progression and metastasis
Source: Oncotarget. 2015 Dec 22;7(4):4680–94. doi: 10.18632/oncotarget.6730 (PMC4826235; doi:10.18632/oncotarget.6730)
Supplement: Supplementary file 1 [file oncotarget-07-4680-s001.pdf]

EGFRvIII/integrin β3 interaction in hypoxic and vitronectin-enriching microenvironment promote GBM progression and metastasis

Supplementary Materials

SUPPLEMENTARY MATERIALS AND METHODS

Microarrays and gene expression analysis

An Agilent Gene Expression array (KangChen Bio-tech Inc., Shanghai, China) of cDNA profiling was applied on U87MG-vector and -EGFRvIII cells (http://www.kangchen.com.cn). The data were normalized in GeneSpring GX by Agilent FE one-color scenario. Differentially expressed genes were identified by fold-changes. Gene Ontology (GO) of the biological process and KEGG pathway enrichment analysis were performed by DAVID http://david.abcc.ncifcrf.gov/) and ranked by *p* values [16].

Primary GBM cultures

Human GBM samples were obtained from the Oncology Hospital affiliated to Harbin Medical University with approval from the Internal Review Board for human research, with both written and verbal consent provided by the patient. Briefly, freshly resected tumors, pathologically confirmed as *de novo* GBM lack of EGFRvIII expression, were minced with a sterile scalpel and chemically dissociated in Trypsin-EDTA (0.25%) for 15–20 mins at 37°C. The dissociated tissue was maintained in DMEM/F12 (1:1) supplemented with 10% FBS and 1% penicillin/streptomycin. The primary GBM culture reached confluence within 2 weeks, was named accordingly (i.e. GBM 11), and stored in the liquid nitrogen at its 2–3 passages. After confirmation of the astrocytic origin by an astrocytic marker (GFAP), the cell culture was ready for further experiments. The establishment of EGFRvIII- or vector- expressing GBM cells was described early.

Wound healing assays

The cells were seeded in 6-well plates at 70% confluence and starved in serum-free medium for 12 h. A "wounding" line was scratched into the monolayer with a sterile 20 µL pipet tip. The monolayer was washed with PBS to remove the detached cells. The serum-free growth medium with/out cilengitide was added to the plates. The cells were then incubated at 37°C for 24 h; the migrated cells were analyzed with a Nikon ECLIPSE Ti-s inverted microscope (Tokyo, Japan) and counted in six randomly selected fields to quantify cell migration rates.

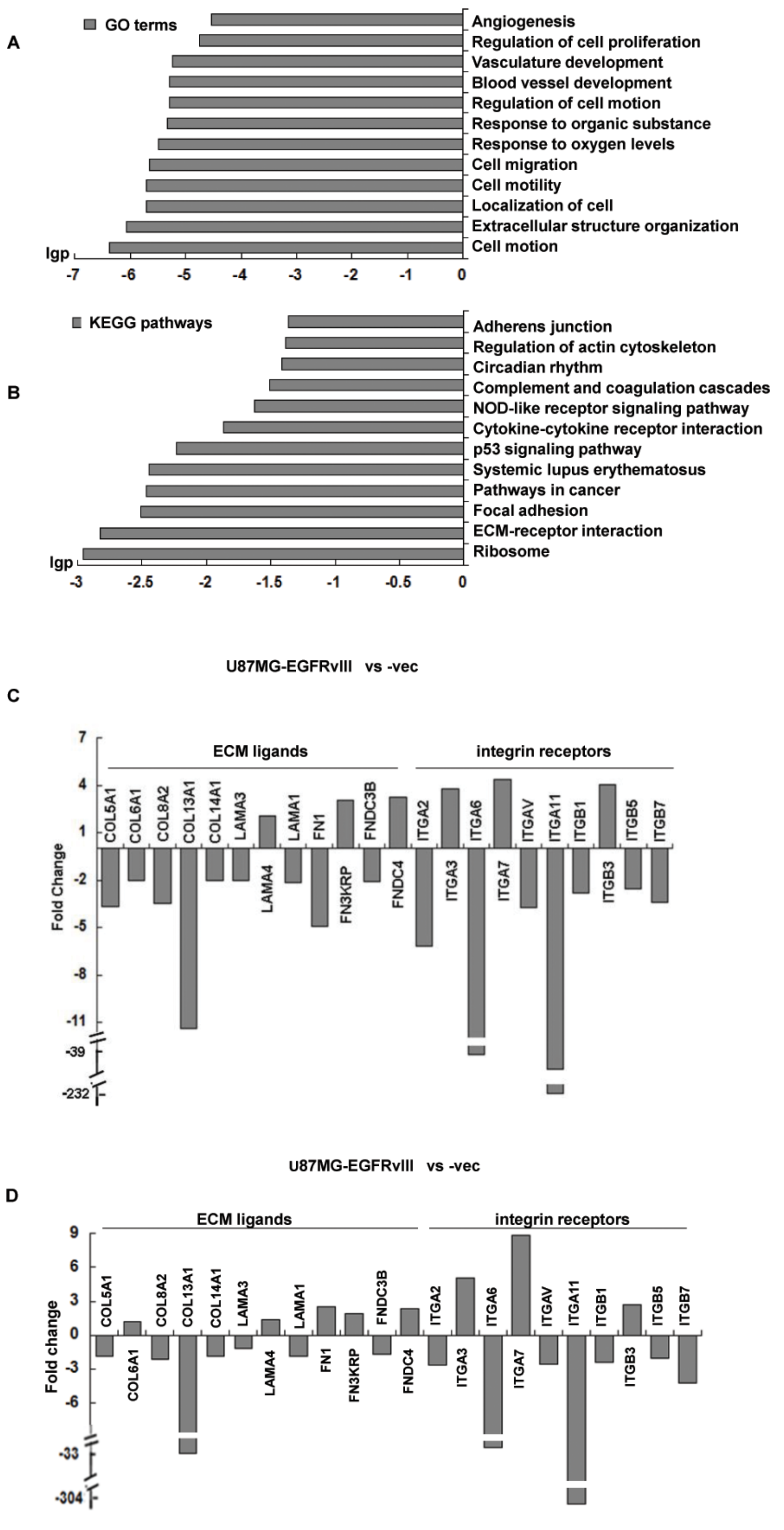

Supplementary Figure S1: Bioinformatics analysis on cDNA profiling of EGFRvIII-expressing GBM cells vs the vector control. Top 12 of the most significant GO terms of the Biological Process (**A**) and top 12 of the most significant KEGG pathways by enrichment study (**B**) ranked by p values. Significant changes (as the fold change) of ECM ligands and integrin receptors at the mRNA level by cDNA profiling (**C**) and the real-time RT-PCR verification (**D**) in U87MG-EGFRvIII GBM cells as compared to that in the vector cells. Representative data were shown by real-time RT-PCR verification. ECM, extracellullar matrix.

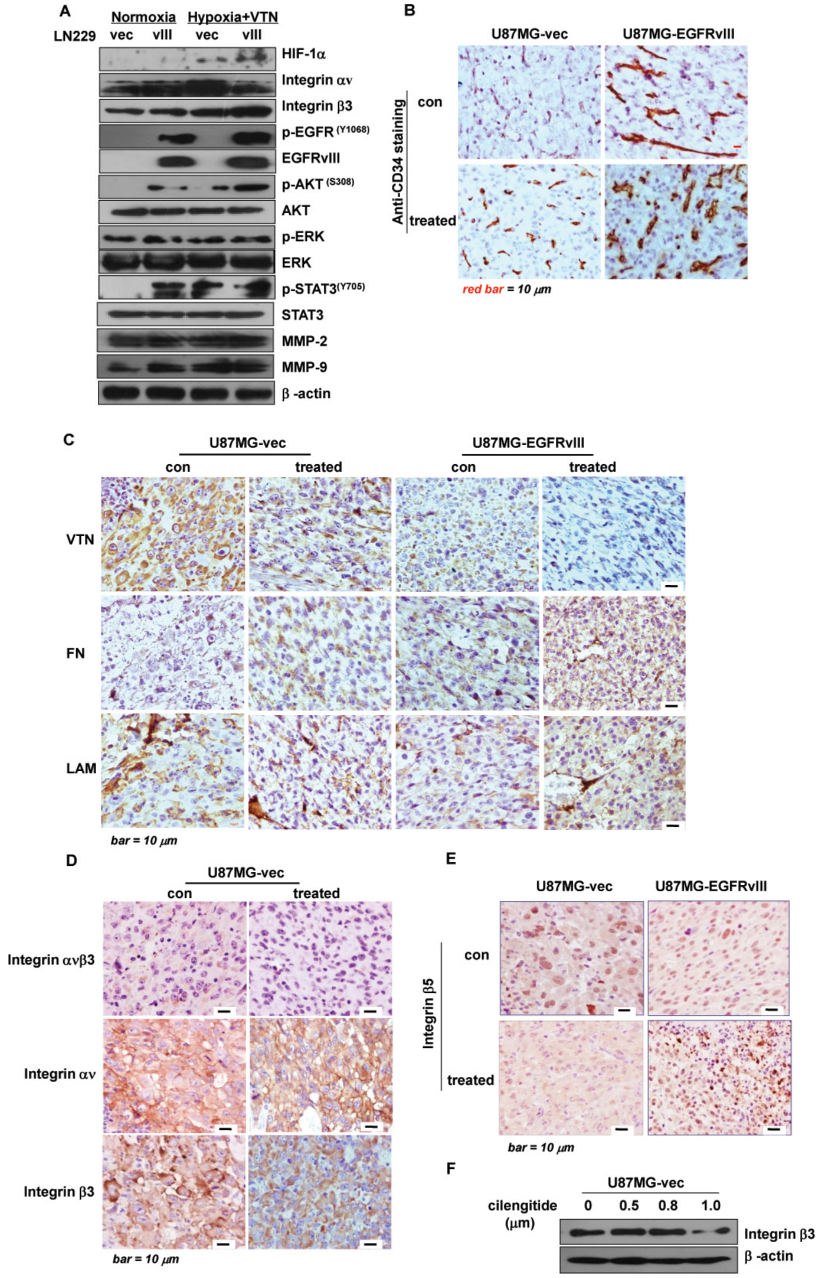

Supplementary Figure S2: Confirmatory experiments with EGFRvIII-expressing GBM and the vector controls. (**A**) LN229-EGFRvIII and vector cells were incubated on normal or hypoxia & vitronectin conditions for 24 h respectively, followed by western blotting analysis on whole-cell lysates for HIF-1α, p-EGFRvIII$^{Y1068\ (145kD)}$, p-ERK1/2$^{T202/Y204}$, p-AKT^{S308}, p-STAT3^{T705}, MMP-2, MMP-9. For normalization purposes membranes were stripped and re-blotted with antibodies for total proteins. (**B, C, D and E**) Immunohistochemistry (IHC) staining with varied antibodies in U87MG-vector or -EGFRvIII xneografts with/out cilengitide treatment. (**F**) Cilengitide treatment less efficiently inhibited the expression of its target integrin β3 in U87MG-vector cells than -EGFRvIII cells as that shown in Figure 7E. Cilengitide was used on U87MG-vector cells for 24 h.

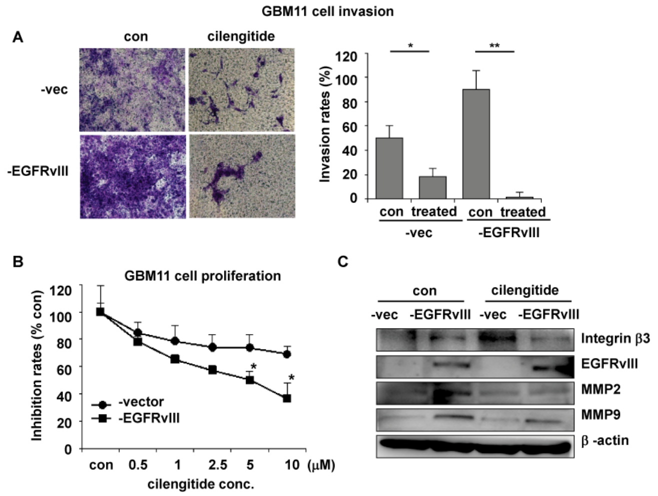

Supplementary Figure S3: Cliengitide preferentially inhibits cell invasion and proliferation in EGFRvIII-expressing GBM cultures *in vitro.* (**A**) Cliengitide treatment attained a greatly better inhibition effect on cell invasion in EGFRvIII-expressing GBM cells than in vector cells in the environment of hypoxia and vitronectin-enrichment. (**B**) In a concentration-dependent manner, cilengitide showed more effective inhibition on cell proliferation in EGFRvIII-expressing GBM cells than in vector cells. (**C**) The treatment more efficiently inhibited the expression of the integrin β3, MMP2, MMP9 in EGFRvIII-expressing GBM than vector control cells. According to IC50 in (B), 5 μM cilengitide was used on GBM 11 cells for 24 h. All experiments were performed independently at least three times. $*p < 0.05$; $**p < 0.01$.
